# Therapeutic plasma exchange: a second‐line treatment for brodifacoum poisoning following an anaphylactoid reaction to vitamin K

**DOI:** 10.1002/ccr3.756

**Published:** 2016-12-18

**Authors:** Ying Deng, Li Qiu

**Affiliations:** ^1^ Department of PediatricsWest China Second UniversityHospital/Key Laboratory of Birth Defects and Related Disease of Women and ChildrenMinistry of EducationSichuan UniversityChengdu610041China

**Keywords:** Anaphylactoid reactions, Brodifacoum poisoning, therapeutic plasma exchange, vitamin K1

## Abstract

Vitamin K1 is the first‐line therapy to brodifacoum poisoning. Anaphylactoid reactions may occur anytime during intravenous administration of vitamin K1 injection. Vitamin K1 must be ceased when anaphylactoid reactions emerge, and therapeutic plasma exchange could be a second‐line option.

## Introduction

Anticoagulants are the most commonly used rodenticides around the world. According to the report of the China Association of Poison Centers, 1567 cases of anticoagulant rodenticide poisonings were reported in 2000 1. Brodifacoum is a highly toxic and long‐acting anticoagulant rodenticide which belongs to the class of second‐generation anticoagulant rodenticides. It induces a deficiency of active vitamin K by inhibiting the epoxide reductase, which can reduce the conversion of vitamin K epoxide to its functional form. The anticoagulant effect of brodifacoum is more than 100 times as potent as warfarin. Therefore, brodifacoum may lead to significant coagulopathy, morbidity, and mortality. The median lethal dose ranges from 0.12 to 0.172 mg/kg via oral ingestion in human 2.

Several extracorporeal methods for poison decontamination have promise in emergency medicine department. Therapeutic plasma exchange (TPE) is a kind of extracorporeal method that plasma‐containing toxicants are removed from the patient and replaced with another fluid (e.g., fresh frozen plasma). It is recommended mainly for neurologic, immunogenic, and hematologic diseases 3. Today, TPE is a promising treatment modality in poisoning cases 4. But there are no reports about the TPE as a method in brodifacoum poisoning.

The most frequently used approach to brodifacoum poisoning is short‐term intravenous and long‐term oral vitamin K1 therapy, along with early utilization of fresh frozen plasma (FFP) or prothrombin complex concentrate (PCC) in the case of active bleeding 2, 5. But anaphylactoid reactions of intravenous VK1 injection are remarkable. According to the State Food and Drug Administration in China, 95.3% of VK1 adverse reactions were associated with the intravenous approach and only 4.7% of adverse reactions were related to other approaches (oral, subcutaneous, intramuscular) 6. When the anaphylactoid reactions happen, the vitamin K1 injection must be halted; what's the alternative treatment for brodifacoum poisoning in these circumstances? There are limited data or guidance about this issue up to now, so we describe a case of a child who developed vitamin K1 anaphylactoid reactions after an exposure to brodifacoum. We succeeded to reverse the coagulopathy via fresh frozen plasma and eliminate the toxicant by TPE.

## Case Details

A female child (2 years and 7 months old) was transferred to our hospital with a chief complaint of ecchymosis and epistaxis. The child's vital sign on administration included temperature of 36.4°C, blood pressure of 83/51 mmHg, pulse rate of 90 beats/min, and respiratory rate of 26 breaths/min. She was well nourished, and her weight was 11 kg. Physical examination revealed moderate epistaxis and ecchymosis scattered in the trunk, limbs, and face. She had a history of exposure to rodenticide. Initial laboratory test results included a PT of >150 sec (reference range, 8.5–14.5 sec), an APTT of 177.4 sec (reference range, 20.4–40.4 sec), a hemoglobin concentration of 91 g/L (reference range, 108–145 g/L),a hematocrit of 26.4% (reference range, 32.7–42.5%), and a positive result of fecal occult blood testing. And the concentration of plasma brodifacoum was 300 ng/mL. Results of liver and renal functional test were within normal limits. She was diagnosed with brodifacoum poisoning. To reverse the coagulopathy, 10 mg intravenous VK1 (1 mL vitamin K1 injection was diluted in 100 mL 5% dextrose, and the speed of intravenous drip was 5 mL/min) and 400 international units PCC were immediately administered. During her hospital stay, she received 10 mg intravenous VK1 daily. Her INR values normalized to 1.17 (reference range, 0.8–1.5) at hospital day 6. Then, she was discharged and continued to receive intravenous VK1 in a community hospital. But 6 days after discharge, she suddenly suffered from fever, shortness of breath, cyanosis, and rash in trunk and limbs after a few minutes of VK1 intravenous infusion (5 mL/min). Her vital signs at that time were blood pressure of 78/46 mmHg, pulse rate of 127 beats/min, respiratory rate of 40 breaths/min, and temperature of 39°C. These symptoms released after the infusion of VK1 was ceased. In the following two times of intravenous VK1 infusion, similar symptoms were found and the doctor had to cease VK1 infusion. This condition was considered to be anaphylactoid reactions, and she was transferred to our hospital to obtain a subsequent treatment. The laboratory test results included a PT of 70.5 sec,an APTT of 54.1 sec, an INR of 6.24, a hemoglobin concentration of 90 g/L,a hematocrit of 26.6%, a serum IgE concentration of 0.6 mg/L, and a Brodifacoum concentration of 50 ng/mL. To reverse the coagulopathy, 150 mL fresh frozen plasma (FFP) was immediately administered via intravenous approach. Her PT decreased to 27.4 sec, APTT decreased to 35.5 sec, INR decreased to 2.4 (reference range, 0.8–1.5) at hospital day 2, and she was treated with 150 mL FFP again. We recommended plasma exchange as the alternative therapy to VK1 after a communication with her parents. She received a plasma exchange treatment every other day, and the plasma volume of each exchange was 500 mL. After three times of plasma exchange, the brodifacoum was not detected in her body. After five times of plasma exchange, the INR value normalized to 1.13 and then, she was discharged. The results of coagulation tests were all within normal limits during the 4‐month follow‐up.

## Discussion

Brodifacoum's molecular weight is 523.42. The elimination of brodifacoum is slow and follows a first‐order kinetic metabolism, with a terminal half‐life ranging from 16 to 270 days in humans 7. The major route of elimination after oral administration is by feces, while only a minor part is by urine 2.

Treatment of brodifacoum poisoning can be challenging when patients had VK1 injection‐induced anaphylactoid reactions. In this case report, we describe both the acute management and the sequential treatment of brodifacoum poisoning when vitamin K1 injection‐induced anaphylactoid reactions happen. This child had some abnormal results of coagulation studies (INR 6.24), which had to be treated. But she also suffered VK1 injection‐induced anaphylactoid reactions, so the intravenous VK1 had to be ceased. Because the oral VK1 was not available at our hospital, the FFP is chosen as an alternative therapy for reversing her coagulation abnormalities. FFP has been used to treat prolonged INR due to vitamin K antagonists. Transfusion of FFP is the preferred treatment method for urgent warfarin reversal (the first‐generation anticoagulant rodenticides) in USA 8. In a review about the management of anticoagulant rodenticide poisoning, the recommended dose is 15–30 mL/kg 9. In the report of King et al. 7, in patients with brodifacoum poisoning, if compatible coagulation abnormalities coincide with serious bleeding, the hemostatic therapy involves immediate treatment with FFP and intravenous VK1.

TPE is effective in eliminating substances with high plasma protein binding capacity (>80%) and low distribution volume (<0.2 L/kg body weight) 10. Brodifacoum is a kind of superwarfarin with a high protein binding affinity (98%~99%) and low distribution volume (0.11–0.15 L/kg) 11, so brodifacoum poisoning is the optimal indication of TPE. The plasma albumin binding with brodifacoum would be replaced by an equal amount of fresh frozen plasma. The estimated plasma volume (EPV) is calculated by 0.07*(1‐HCT)*weight. In this case, the EPV was about 500 mL [0.07*(1‐0.26)*11]. The general recommendation for TPE is that each exchange consisting of 1–1.5 plasma volumes be performed every 2 or 3 days, and a total of three to five procedures for most cases 12. We treated this child with TPE 500 mL per procedure, and the plasma levels of brodifacoum decreased from 50 to 0 ng/mL after three procedures of TPE. After five procedures of TPE, she was discharged from hospital and was followed up for 4 months. Besides the usage in brodifacoum poisoning, the TPE can also be used in other poisoning cases, and many doctors have elucidated the safety and effectiveness of plasmapheresis or TPE, such as in amitriptyline, amlodipine, theophylline, carbamazepine, diltiazem, verapamil, propranolol, L‐thyroxine, and mercury poisoning cases 4, 13, 14. Dişel et al. 15 claimed in their study that TPE may be a promising toxicant elimination and treatment technique in poisoned patients when implemented in selected cases.

In our report, we consider TPE as a second‐line therapy following anaphylactoid reactions to VK1. The child recovered and showed no adverse reactions after receiving an amount of 2500 mL plasma exchange (five procedures). We consider the TPE to be an effective and safe treatment for brodifacoum poisoning when VK1 injection‐induced anaphylactoid reactions happen. FFP is a fine option to reverse the coagulation abnormalities and bleeding. In a review of King et al. 7, the reported length of oral VK1 treatment in Brodifacoum poisoning cases ranged from 28 to 330 days, while in our report, using TPE, the child has no need for long‐time VK1 treatment. As everyone knows, treatment with oral VK1 therapy is prolonged. The compliance of patients is another dilemma for both the patients and the doctors.

To the author's knowledge, this is the first case report about TPE in treating brodifacoum poisoning following VK1 injection‐induced anaphylactoid reactions. Our report represents a scientific description of personalized medicine. TPE is an effective and safe approach, and we would recommend its use in special cases in which patients cannot tolerate VK1 or they have severe coagulopathy. More robust scientific investigations are needed to ascertain the full benefits of TPE in the treatment of superwarfarin poisoning.

## Conclusions

A child aged 2 years and 7 months diagnosed with brodifacoum poisoning was successfully treated with TPE. If anaphylactoid reactions emerge and VK1 has to be ceased, TPE could be a second‐line option to brodifacoum ‐poisoning.

## Conflict of Interest

The authors have no conflict of interests to declare.

## Authorship

YD: wrote the first draft of the manuscript.LQ: gave advices on this manuscript and helped collect data.
